# Association of transforming growth factor-β1 gene C-509T and T869C polymorphisms with atherosclerotic cerebral infarction in the Chinese: a case-control study

**DOI:** 10.1186/1476-511X-10-100

**Published:** 2011-06-16

**Authors:** Zhongxing Peng, Lixuan Zhan, Shengqiang Chen, En Xu

**Affiliations:** 1 Institute of Neurosciences, The Second Affiliated Hospital of Guangzhou Medical College, Key Laboratory of Neurogenetics and Channelopathies of Guangdong Province and the Ministry of Education of China, Guangzhou 510260, P.R. China; 2 Department of Neurology, the First Affiliated Hospital of Guangdong Pharmaceutical College, Guangzhou 510080, P.R. China

## Abstract

**Background:**

Transforming growth factor-β1 (TGF-β1) is a multifunctional cytokine involved in inflammation and pathogenesis of atherosclerosis. There is scant information on the relation between variations within the TGF-β1 gene polymorphisms and risks of ischemic cerebrovascular diseases. Therefore, this case-controlled study was carried out to investigate the possible association of the TGF-β1 gene C-509T and T869C polymorphisms, and their combined genotypes with the risk of atherosclerotic cerebral infarction (CI) in the Chinese population.

**Results:**

We recruited 164 CI patients and 167 healthy control subjects who were frequency-matched for age and gender. The frequencies of the -509TT genotype and T allele gene were significantly higher in the CI group (*P *= 0.007, *P *= 0.006). The frequencies of +869CC genotype and C allele were higher in the CI group (*P *= 0.002, *P *= 0.004). In the CI group, the individuals with -509TT genotype had a significantly higher level of plasma triglyceride (TG) (*P *= 0.017). +869CC genotype correlated significantly with higher level of plasma low density lipoprotein cholesterol (LDL-c) in the CI group (*P *= 0.015). With haplotype analysis, the frequency of the -509T/+869C combined genotype was significantly higher in the CI group than in controls (*P *< 0.001).

**Conclusions:**

Our study suggests that C-509T and T869C gene polymorphisms in TGF-β1 may be a critical risk factor of genetic susceptibility to CI in the Chinese population.

## Background

The etiology of atherosclerotic cerebral infarction (CI) is complicated as genetic and environmental factors are all involved. Epidemiological and animal studies have showed that genetic factors determine the development of cerebrovascular disease (CVD) [1,2]. Significant researches have been conducted to establish the relationship between the functional variants of a variety of genes and the risk of stroke in different ethnic groups across the world [3]. These include the endothelial nitric oxide synthase gene[4], angiotensin-converting enzyme gene[5], genes associated with lipid metabolism [e.g. lipoprotein lipase, and paraoxonase-1] [6], thrombosis and coagulation (e.g. factor V Leiden, fibrinogen, and prothombin), Apolipoprotein E, 5.10, Methylenetetrahydrofolate reductase, and Protein Z gene, etc. Inflammation plays an important role in the pathogenesis of atherosclerosis and arterial thrombosis [7]. Variations in the genetics of the inflammatory system may increase the risk of diseases, such as ischemic heart disease [8]. Transforming growth factor-β1 (TGF-β1) is a multifunctional cytokine involved in inducing cell differentiation, vascularization, and migration [9]. Compelling evidences showed that TGF-β1 is involved in pathologic states such as inflammation processes, atherosclerosis, and restenotic lesions [8,10,11]. However, the roles of TGF-β1 on cardiovascular and cerebrovascular diseases are controversial. Numerous studies have demonstrated that the involvement of TGF-β1 in atherosclerosis plays a key role by inhibiting inflammation and increasing atherosclerotic plaque stabilization, and hence appears to be anti-atherogenic [12-14]. However, recent studies demonstrated that TGF-β1 is associated with vascular stenosis and thrombogenesis [15,16] by promoting fibrosis and inhibiting endothelial regeneration [17,18] and hence is a pro-atherogenic factor.

The TGF-β1 gene is located on chromosome 19 (q13.1-13.3). Six commonly known polymorphisms were found in the TGF-β1 gene, including C-988A (rs1800820), G-800A (rs1800468), C-509T (rs1800469), T869C (rs1982073; Leu10/Pro10; T29->C), G915C (rs1800471), and C11929T (Thr263Ile; rs1800472) [19-21]. The previous studies attempted to determine whether naturally occurring polymorphisms in the TGF-β1 gene affect TGF-β1 expression [20,22,23]. A C-to-T single nucleotide polymorphism (SNP) at position-509 relative to the first major transcription start site was found to be differentially related to transcription factor binding to the TGF-β1 promoter, transcriptional activity of TGF-β1, and TGF-β1 plasma concentration [20,24]. The T869C SNP is located at position 29 of the translated sequence of TGF-β1 and gives rise to amino acid substitutions at position 10 (Leu10Pro) in the signal peptide of TGF-β1. The T869C SNP was reported to influence steady-state concentrations of TGF-β1 mRNA in peripheral blood mononuclear cells and serum levels of TGF-β1 [22,25]. Also, *in vitro *experiments have reported that the Pro10Leu substitution in the signal peptide affected significantly the secretion of the TGF-β1 protein in HeLa cells [23]. Up to now, several TGF-β1 gene mutation loci have been detected to investigate the alternation of lipid levels in plasma and the development of coronary artery disease (CAD) and atherosclerotic cerebral infarction. Of these, C-509T and T869C gene polymorphisms are common. The C-509T gene polymorphism of promoter region of TGF-β1 gene is associated with the level of plasma LDL-c [26] and myocardial infarction (MI) in the German white population [27] and young Italian population [28], but the corresponding study in the Dutch [29] was negative. The T869C gene polymorphism in exon 1 of the TGF-β1 gene was also reported to be associated with elevated serum lipoprotein (a) in renal transplant recipients [30] and MI in Japanese [22] and young Italian population [28], but the corresponding studies in other races [29,31,32] were negative. Sie et al. [29] reported that the C-509T and T869C gene polymorphisms were associated with CI.

Recent studies have demonstrated the critical role of the TGF-β1 signaling pathway in atherosclerosis [33]. Furthermore, the influence of polymorphisms of TGF-β1 gene on inter-individual variation in atherosclerotic vascular disease inspired us to postulate that TGF-β1 C-509T and T869C gene polymorphisms may influence CI. Therefore, the objective of the present research was to clarify the relationship among these two TGF-β1 gene polymorphisms, plasma lipid profile, and CI, and to explore whether there is any combined genotype that plays a cardinal role in the process of CI.

## Methods

### Subjects

The study recruited 164 CI patients (100 male and 64 female, aged 42-76 years), who were admitted to Department of Neurology of the Second Affiliated Hospital of Guangzhou Medical College (44 patients) and Department of Neurology of the First Affiliated Hospital of Guangdong Pharmaceutical College (120 patients), and 167 healthy subjects (95 male and 72 female, aged 52-79 years) who had no history or family history of CVDs as controls.

All subjects enrolled in our study were Chinese with similar dietary pattern, excluding those who had gastrointestinal, severe hepatic, biliary, renal, thyroid diseases, autoimmune diseases, diabetes mellitus, tumor, or malnutrition, and those who had accepted organ transplantation, and one vegetarian. None of them was on any lipid-affected drugs prior to enrollment.

All patients presented clinical characteristics of CI in anterior circulation, confirmed by cranial computed tomography or magnetic resonance imaging/angiography, or both. Color Doppler carotid artery ultrasonography was performed for all CI patients. Detailed history of patients, physical examination, electrocardiography (ECG), prolonged cardiac monitoring (Holter) and transthoracic echocardiography (TTE) were used to exclude cardioembolic stroke. Also excluded were patients who had arthritis, hematopathy, vascular malformation, asymptomatic CI, hemorrhagic infarction and/or intracranial hemorrhage. All the experiments on the subjects were conducted in accordance with the Declaration of Helsinki. Besides, the study was approved by the Hospital Institutional Ethics Committee, and written informed consent was obtained from all study participants.

### Plasma lipid measurements

Blood samples for the lipid measurements were withdrawn from subjects after an overnight fast. Plasma levels of triglycerides (TG), total cholesterol (TC), high density lipoprotein (HDL-c), and low density lipoprotein cholesterol (LDL-c) were measured by Hitachi 7600 automatic analyzer (Hitachi Instruments Corporation, Tokyo, Japan).

### DNA isolation and PCR amplification

Genomic DNA was extracted from peripheral blood leukocytes by the phenol/chloroform method. The primer sets (SBS Genetech, Beijing, China) were based on previously published information [31,34]: C-509T, forward primer: 5'-CAG ACT CTA GAG ACT GTC AG-3' and reverse primer: 5'-GTC ACC AGA GAA AGA GGA C-3'; T869C, forward primer: 5'-ACC ACA CCA GCC CTG TTC GC-3' and reverse primer: 5'-AGT AGC CAC AGC AGC GGT AGC AGC TGC-3'. The PCR amplification of C-509T was performed in a total volume of 30 μl mixture containing: 3 μl (150 ng) genomic DNA, 3 μl 10× buffer solution, 0.5 μl 2.5 U *Taq*DNA polymerase (Shenergy Biocolor Bioscience & Technology Company, Shanghai, China), 1 μl (20 pmol) of each primer, and 3 μl (200 μmol/l) of each deoxynucleotide triphosphate. The PCR conditions were as follows: initial denaturations at 93°C for 5 min were followed by 30 cycles of denaturations at 93°C for 45 s, annealing at 54°C for 45 s, and extension at 72°C for 45 s, with final extension at 72°C for 7 min. Genomic DNA 2 μl (100 ng) of T869C was used for a 50 μl PCR reaction containing 10 μl 5×buffer solution, 1 μl (3 U/μl) *Taq*DNA polymerase (Shenergy Biocolor Bioscience & Technology Company), 1 μl (10 pmol) of each primer, and 1 μl (10 mM) of each deoxynucleotide triphosphate. The PCR conditions were as follows: initial denaturations at 93°C for 5 min were followed by 40 cycles of denaturations at 93°C for 30 s, annealing at 55°C for 45 s, and extension at 72°C for 45 s, with final extension at 72°C for 7 min.

### TGF-β1 gene polymorphism analysis

Digestion of PCR product was performed by addition of 1 μl respective restriction enzyme (C-509T: Eco81I; T869C: Pst Ι (TAKARA Biotechnology Co., Ltd., Dalian, China)) to 10 μl of PCR products in 2 μl 10 × buffer solution, centrifuged for 2 min at the speed of 5000 rounds/min and bathed in aqueous environment at 37°C overnight. The resulting fragments were resolved by electrophoresis (80 V, 60 min) on 2.5% agarose gel and visualized under UV light.

### Direct sequencing of DNA fragment

Direct sequencing of double-stranded DNA fragment was performed by using Beckman DNA sequencer (Beckman Coulter, Inc, Fullerton, CA, USA).

### Statistical analysis

Measurement data were summarized as mean ± standard deviation (S.D.) and compared with two-sample *t*-test. Enumeration count data were summarized as number (%) and compared with chi-square test (*x*^*2*^-test). To assess the Hardy-Weinberg equilibrium for the C-509T and T869C genotype distribution, *x*^*2 *^analysis was performed. The odds ratios (ORs) and 95% CI were used to assess the strength of the relationship in the genotype and allele distribution of polymorphisms C-509T and T869C between the CI group and controls. Haplotype frequencies were estimated in the C-509T and T869C polymorphisms, and the ORs and 95% CI were also used to estimate the haplotype association risk between the CI group and controls. All tests were two-side and a *P *value < 0.05 was considered statistically significant. Statistical analyses were performed with the Statistical Package for Social Sciences for Windows, version 11.5 (SPSS, Inc, Chicago, IL, USA).

## Results

Clinical characteristics of the 331 participants (164 CI patients and 167 control subjects) are presented in Table 1. The risk factors examined (e.g. CAD, hypertension, and smoker) were significantly more common in the CI group than in controls. Systolic blood pressure (SBP), diastolic blood pressure (DBP), and plasma level of TG tended to be higher, while HDL-C was lower in CI patients than in controls at admission.

**Table 1:** Characteristic of Controls and CI Patients

Characteristic	Controls	CI group	*P *value
n	167	164	
Age, years	63.68 ± 6.65	64.87 ± 6.16	0.094
Female, %	72(43.1)	64(39.0)	0.450
BMI, Kg/m^2^	22.60 ± 1.90	22.92 ± 1.89	0.118
SBP, mmHg	127.62 ± 12.33	148.08 ± 23.71	<0.001
DBP, mmHg	75.12 ± 6.20	85.63 ± 13.56	<0.001
CAD, %	5(3.0)	15(9.1)	0.019
TG, mmol/L	1.28 ± 0.41	1.64 ± 0.61	<0.001
TC, mmol/L	5.17 ± 0.86	5.13 ± 1.12	0.670
HDL-c, mmol/L	1.38 ± 0.35	1.26 ± 0.34	0.002
LDL-c, mmol/L	3.08 ± 0.73	3.14 ± 0.97	0.493
Hypertension, %	14(8.4)	93(56.7)	<0.001
Smoker, %	45(26.9)	67(40.9)	0.008
Drinker, %	44(26.3)	36(22.0)	0.263

Data are presented as means ± S.D. or n (%). Student's t test and χ2 test were used to compare the values of controls and CI patients. BMI, body mass index; SBP, systolic blood pressure; DBP, diastolic blood pressure; CAD, coronary artery disease; TG, triglyceride; TC, total cholesterol; HDL-c, high density lipoprotein cholesterol; LDL-c, low density lipoprotein cholesterol

All genotype and allele proportions were in Hardy-Weinberg equilibrium in both controls and CI group.

The genotype distribution and the allele frequency for C-509T and T869C gene polymorphisms are summarized in Table 2. A preliminary case-control analysis of the population revealed significant differences in the allele and genotype frequency of both C-509T and T869C site polymorphisms. The frequency of T allele of C-509T was significantly higher in the CI group than in controls (*P *< 0.006). The distribution of C-509T gene polymorphism demonstrated that CI patients had higher TT frequency compared with controls (*P *< 0.007). The frequency of C allele of T869C was significantly higher in the CI group than in controls (*P *< 0.004). The distribution of T869C gene polymorphism demonstrated that CI patients had higher CC frequency compared with controls (*P *< 0.002).

**Table 2:** Genotype Distributions and allele Frequencies of the C-509T and T 869C

		Genotype, n (%)		Allele Frequency
-509C/T	TT	CT	CC	T Allele (%)
Controls (n = 167)	59(35.3)	79(47.3)	29(17.4)	59.0
CI Group (n = 164)	82(50.0)	63(38.4)	19(11.6)	69.2
OR	1.83	0.70	0.63	1.56
95% CI	1.18-2.85	0.45-1.08	0.33-1.16	1.14-2.15
*P *value	0.007	0.135	0.102	0.006

869T/C	TT	TC	CC	C Allele (%)
Controls (n = 167)	46 (27.5)	86(51.5)	35(21.0)	46.7
CI Group (n = 164)	34(20.7)	70(42.7)	60(36.6)	57.9
OR	0.69	0.70	2.18	1.57
95% CI	0.41-1.14	0.46-1.08	1.33-3.55	1.16-2.14
*P *value	0.148	0.108	0.002	0.004

As it has been shown that cholesterol inhibits the activation of TGF-β1 [35] and that this could be an important mechanism in the initiation and progression of atherosclerosis, a possible interaction was tested between lipid levels of plasma and the TGF-β1 C-509T and T869C gene polymorphisms on the risk of CI. The C-509T and T869C mutations did not correlate significantly with levels of plasma TG, TC, HDL-c, and LDL-c in controls (data not shown). In the CI group, the individuals with -509TT genotype had a significantly higher level of plasma TG. Compared with 869CT and 869TT mutation, 869CC genotype correlated with a significantly higher level of plasma LDL-c. The relationships between genotypes and levels of plasma lipids in the CI group are shown in Table 3.

**Table 3:** Plasma Concentrations of TG, TC, HDL-c, and LDL-c in Various Genotypes of the C-509T and T 869C in CI Group

Genotype	TG(mmol/L)	TC(mmol/L)	HDL-c(mmol/L)	LDL-c(mmol/L)
-509C/T				
TT	1.79 ± 0.56	5.28 ± 1.17	1.27 ± 0.35	3.26 ± 0.95
CT	1.53 ± 0.67	4.91 ± 0.94	1.22 ± 0.31	3.09 ± 0.80
CC	1.48 ± 0.43	5.17 ± 1.41	1.34 ± 0.42	3.11 ± 1.20
*P *value	0.017	0.133	0.378	0.513

869T/C				
TT	1.58 ± 0.68	4.92 ± 1.35	1.28 ± 0.36	2.94 ± 0.95
TC	1.61 ± 0.66	4.98 ± 1.00	1.26 ± 0.34	3.01 ± 0.86
CC	1.57 ± 0.54	5.33 ± 1.19	1.16 ± 0.24	3.43 ± 0.99
*P *value	0.953	0.134	0.100	0.015

Values are mean ± S.D.

To address the possibility of combined effects of the TGF-β1 single nucleotide polymorphisms (SNPs), the possible associations between SNP-based haplotypes and CI were examined. The distribution of combined genotype is presented in Table 4. The frequency of T/C (-509T and 869C) combined genotypes was higher in the CI group than in controls (*P *< 0.001). The frequency of C/T (-509C and 869T) combined genotype was lower in the CI group than in control subjects (*P *= 0.010).

**Table 4:** Combined Genotype Distributions of the C-509T and T 869C in Controls and CI Group

		Combined Genotype, n (%)		
-509C/T/869T/C	-509T/869T	-509T/869C	-509 C/869T	-509 C/869C
Controls (n = 167)	15(9.0)	20(12.0)	125(74.8)	7(4.2)
CI group (n = 164)	10(6.1)	50(30.5)	101(61.6)	3(1.8)
OR	0.66	3.22	0.54	0.43
95% CI	0.29-1.519	1.82-5.72	0.34-0.86	0.11-1.68
*P *Value	0.321	<0.001	0.010	0.209

**Figure 1:**
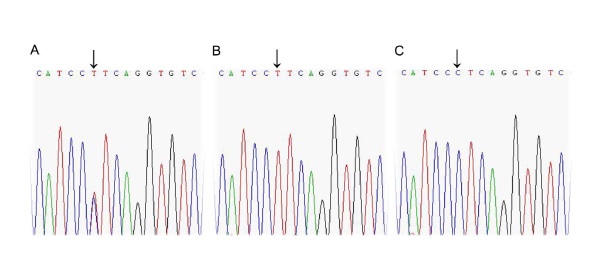
**Direct sequencing of genomic DNA in the vicinity of -509C/T polymorphism of TGF-β1 gene**. Representative results implicate: heterozygous subject (A); subject homozygous for C (B); subject homozygous for T (C)

**Figure 2:**
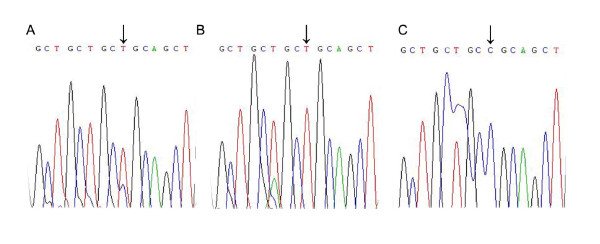
**Direct sequencing of genomic DNA in the vicinity of 869T/C polymorphism of TGF-β1 gene**. Representative results implicate: heterozygous subject (A); subject homozygous for T (B); subject homozygous for C (C)

Figure 1 and Figure 2 show direct sequencing of genomic DNA in the vicinity of C-509T and T869C loci of TGF-β1 gene.

## Discussion

The present study was the first time to evaluate the relationships between SNPs at the TGF-β1 loci and CI and lipid levels of plasma in the Chinese population. The major findings of our study were as follows: (1) the -509T and the 869C allele are more frequent in CI patients than in controls. (2) In the CI group, the individuals with -509TT genotype had a significantly higher level of plasma TG, and +869CC genotype correlated with significantly higher level of plasma LDL-c in the CI group. (3) The frequency of the -509T/+869C combined genotype was significantly higher in the CI group than in controls.

The C-509T polymorphism of the TGF-β1 gene is located in the promoter region which is relative to the first major transcription start site and was found to be related to transcriptional activity of TGF-β1, and TGF-β1 plasma concentration [20]. It has been reported that the homozygote for C in the C-509T allele of TGF-β1 is associated with higher mRNA expression [25] and also with higher serum concentration of TGF-β1 [22]. In the study of Oda et al [36], it was found that the T-allele at C-509T of TGF-β1 is a risk factor for atherogenesis of the intracranial arteries in the Japanese elderly. Sie's [29] study showed that subjects with the -509CT genotype and T-allele had a significantly increased risk of ischemic stroke and overall stroke, including ischemic stroke, hemorrhagic stroke and unspecified stroke. Similar to Sie's [29] study, the frequency of the T allele in CI patients was significantly higher than that in control subjects, and the subjects in CI group had higher TT frequency compared with controls in this study.

The T869C polymorphism is located in the signal peptide sequence; this sequence is involved in the export of synthesized proteins across membranes of the endoplasmic reticulum. Signal peptides exhibit a unity of function despite having highly diverse sequences; however, they all comprise three regions: a positively charged N-terminal region, a central hydrophobic core, and a polar C-terminal region. The T869C polymorphism is located in the hydrophobic core. Different classes of signal sequence mutations changing one amino acid to another and affecting export efficiency have been described [37], and the importance of a change in charge in the hydrophobic core has been stressed. The T869C polymorphism was reported to influence steady-state concentrations of TGF-β1 mRNA in peripheral blood mononuclear cells and serum levels of TGF-β1 [22,25]. For the T869C polymorphism, the association between the polymorphism and risk of stroke in the overall stroke group was found to be somewhat stronger than in the ischemic stroke group in the study of Sie et al [29]. However, in our study, a significantly higher CC frequency in the CI group was found compared with controls. Different genetic backgrounds between whites and Chinese may explain, at least in part, the disparate findings of the studies. Heterogeneities in the prevalence of conventional risk factors for CI, including CAD, arterial hypertension, hypercholesterolemia, and cigarette smoking may have also contributed to the divergent results between the study populations.

To date, the relationship between TGF-β1 and lipid metabolism was not completely understood. Previously, TGF-β has been demonstrated to influence the expression of lipoproteins and regulate cholesterol metabolic processes [38]. Also, TGF-β1 inhibits LPL mRNA expression, protein levels and enzymatic activity [39], and hence may influence the plasma levels of lipid. On the other hand, the study of Chen et al [35] found that cholesterol, both in free and complex forms (e.g. LDL-c), suppresses TGF-β responsiveness in vascular cells. Moreover, cholesterol-lowering agents and cholesterol-depleting agents enhance TGF-β responsiveness. However, to our knowledge, only a few studies have been published on the association of the TGF-β1 polymorphisms and lipid levels of plasma. Nordström et al indicated in their study that the absence of the C allele of C-509T was associated with an increased level of serum LDL-c in the adolescent females [26]. Whereas, in our study, we observed that the plasma levels of TG were higher in patients with the -509TT genotype than in subjects with the CC or TC genotype in the CI group. Besides, we found that the plasma levels of LDL-c were higher in CI patients with the 869CC genotype than in subjects with the TT or TC genotype. Taking together the positive effects of the TGF-β1 C-509T and T869C gene polymorphisms on the CI, the present study is the first to suggest that lipid levels of plasma and CI in the Chinese population are related to polymorphism of the same gene. These results suggest that TGF-β1 gene polymorphisms effect on the CI, which may partly be attributed to the regulation of lipid levels of plasma.

A high degree of linkage disequilibrium was observed between pairs of the C-509T and T869C SNPs in white populations [31]. In these regard, further haplotype analysis of the two polymorphisms was conducted, which is more useful for the identification of predisposing genes of complicated diseases [40]. Our results demonstrated that mutant haplotypes TC (-509T and +869C) were associated with a higher risk for developing CI. The risk of CI occurring in subjects carrying this haplotype was 3.22-fold higher (*P *< 0.001) in comparison with those without the TC (-509T and +869C) haplotype. This finding implies that carrying the TC (-509T and +869C) haplotype is a risk factor independently modifying individual susceptibility to the development of CI in the Chinese population. Common complex human diseases, such as stroke, are thought to be under the control of many genes that contribute modest individual effects, and TGF-β1 may act in concert with or independently from other stroke susceptibility loci.

## Conclusion

Our study suggests that the -509T and the 869C allele is more frequent in CI patients than in controls. A significant association was found between the C-509T and T869C gene polymorphisms and risk of CI. The data also provides strong evidence of an association between polymorphisms in the TGF-β1 gene with lipid levels. Moreover, -509T/+869C combined genotype mutation of the TGF-β1 gene are relevant to CI. The C-509T and T869C polymorphisms are the risk factors for CI, indicating that the TGF-β1 polymorphism may remain a useful genetic marker for predicting CI risk in the Chinese. These results warrant further studies, with larger numbers, to consolidate these findings, to investigate the functional effects of these polymorphisms and to study the underlying pathophysiological mechanism.

## List of Abbreviations

CAD: coronary artery disease; CI: cerebral infarction; CVD: cerebrovascular disease; DBP: diastolic blood pressure; ECG: electrocardiography; HDL-c: high density lipoprotein; LDL-c: low density lipoprotein cholesterol; MI: myocardial infarction; SBP: systolic blood pressure; SNP: single nucleotide polymorphism; TC: total cholesterol; TG: triglyceride; TGF-β1: transforming growth factor-β1; TTE: transthoracic echocardiography.

## Competing interests

The authors declare that they have no competing interests.

## Authors' contributions

Cases were recruited by PZ, ZL and XE. Genomic DNA was extracted by PZ and ZL. The PCR amplification was performed by PZ and CS. Polymerase chain reaction-restriction fragment length polymorphisms technique was performed by PZ and CS. Data acquisition, analysis and manuscript preparation were performed by PZ, ZL and XE. All authors have approved this version. XE takes full responsibility for the clinical and experimental data, the analyses and interpretation, and the conduction of research.
